# Extrachromosomal HPV-16 LCR transcriptional activation by HDACi opposed by cellular differentiation and DNA integration

**DOI:** 10.18632/oncotarget.12263

**Published:** 2016-09-26

**Authors:** Ekaterina Dimitrova Bojilova, Christine Weyn, Marie-Hélène Antoine, Véronique Fontaine

**Affiliations:** ^1^ Université Libre de Bruxelles (ULB), Faculty of Pharmacy, Unit of Pharmaceutical Microbiology and Hygiene, 1050 Brussels, Belgium; ^2^ Université Libre de Bruxelles (ULB) Faculty of Medicine, Laboratory of Experimental Hormonology, 1070 Brussels, Belgium

**Keywords:** human papillomavirus, histone deacetylase inhibitor, transcription, integration, differentiation

## Abstract

Histone deacetylase inhibitors (HDACi) have been shown to render HPV-carrying cells susceptible to intrinsic and extrinsic apoptotic signals. As such, these epigenetic drugs have entered clinical trials in the effort to treat cervical cancer. Here, we studied the effect of common HDACi, with an emphasis on Trichostatin A (TSA), on the transcriptional activity of the HPV-16 Long Control Region (LCR) in order to better understand the impact of these agents in the context of the HPV life cycle and infection. HDACi strongly induced transcription of the *firefly* luciferase reporter gene under the control of the HPV-16 LCR in a variety of cell lines. In the HaCaT keratinocyte cell line undergoing differentiation induced by TSA, we observed a reduction in LCR-controlled transcription. Three major AP-1 binding sites in the HPV-16 LCR are involved in the regulation by TSA. However, whatever the status of differentiation of the HaCaT cells, TSA induced integration of extra-chromosomal transfected DNA into the cellular genome. Although these data suggest caution using HDACi in the treatment of HR HPV infection, further *in vivo* studies are necessary to better assess the risk.

## INTRODUCTION

More than 120 human papillomaviruses (HPV) have been described and shown to be globally distributed. They seem to coexist with their host over a long period, sometimes in a latent life cycle, as suggested by the wide variety of different types detected at random sites of healthy skin [1, 2]. Persistent genital infections by some specific types of the alpha-papillomavirus genus, called high-risk HPV (HR-HPV), have been associated with a high risk of cervical malignant progression, most notably HPV type 16 (HPV-16) [3].

Studies of the ecto-endocervical junctions revealed residual embryonic cell populations able to differentiate and characterized by vulnerability to undergo neoplastic transformation [4]. Mirkovic *et al*. suggest that these cells, when infected by HR HPV, might comprise a nidus for early lesion development [5]. Following viral entry into such cells, the viral particle is uncoated, DNA is trafficked to the nucleus where viral episomal replication can occur, replicating HPV genomes to about a 100 episomal copies per cell [6]. Integration of the viral genome into the host DNA can take place, especially in case of long-term maintenance in the basal epithelium [3].

Viral episomes are retained in the nucleus and coordinately partitioned into daughter cells during mitotic division. The ability to maintain a stable copy number is an attribute of all HPVs, and has been thought to require early viral proteins such as the oncogenic E6 and E7 [7, 8], the E1 helicase and the E2 protein binding the mitotic chromosome-associated Brd4 protein [9, 10].

Reversible acetylation of histones is a crucial event in the regulation of gene expression [11]. This process is orchestrated by histone acetyltransferases (HATs) and histone deacetylases (HDACs). Acetylation at particular lysine residues on histones is associated with a relaxed, open chromatin configuration and therefore increased accessibility of transcription factors to their binding sites on target genes. Conversely, deacetylation of these lysines by HDACs leads to a more condensed chromatin structure around promoters and cis-elements, and therefore – to silencing of transcription. Thus, inhibitors of HDACs (HDACi) perturb the cellular transcription environment to a significant extent. HDACi have been shown to alter the expression of about 2% of cellular genes, including genes controlling the cell cycle and apoptosis, such as p21(waf1), c-myc, and p53 [12–15]. A study by Choi *et al*. has shown that acetylation of histone H4 is markedly increased after treatment with Trichostatin A (TSA), the most potent HDACi known to date [16].

Several studies have shown that HDACi induce apoptosis in HPV-positive cell lines [17, 18] and arrest their growth independently of E6/E7 oncogene expression [19, 20]. Consequently, HDACi could be attractive drugs for the treatment of HPV infection and entered clinical trials. On the other hand, HDACi are also well-known to activate many viral promoters, such as that of HIV-1, neurotropic JC polyomavirus, Epstein-Barr virus (EBV), Kaposi virus and even HPV-11 [21–25]. As such, they have emerged as potential drugs to, for example, “shock and kill” latent HIV-1 reservoirs not susceptible to combinatory antiretroviral therapy (CART) [22, 26–29].

Although the chromatin structure of the intrachromosomal HPV-16 LCR has been reported to repress its transcriptional activity [30], little is known on the impact of HDACi on episomal HPV-16 LCR [31]. As HPV infections and multiple co-infections are common and ubiquitous, we investigated whether HDACi can also enhance HPV-16 long-control-region (LCR)-induced transcription and the impact of HDACi on the integration of extra-chromosomal DNA into host DNA.

## RESULTS

### Histone Deacetylase Inhibitors (HDACi) induce the HPV-16 Long Control Region (LCR) transcriptional activity in transformed human cell lines

**Figure 1:**
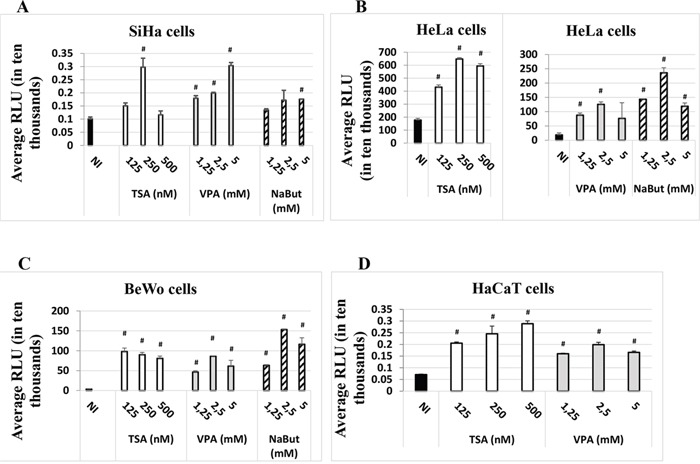
HDACi induce the expression of the *luc* gene under the control of the HPV-16 LCR in the SiHa **A.** HeLa, **B.** BeWo **C.** and HaCaT **D.** cell lines. Cells were transfected with the HPV-16 pWtLCRluc plasmid. HDACi TSA (white), VPA (grey) or NaBut (striped) were added at the time of transfection at the concentrations indicated. NI: non-HDACi-treated transfected control cells (black). Twenty-four hours post-transfection, cells were lysed and intracellular protein luminescence (represented as average RLU units on the graphs) was read on a luminometer. The results are the average of at least three independent experiments for each cell line indicated. Bars represent standard deviations. Hash marks above standard deviations represent statistically significant data, compared to NI.

We investigated the capacity of the HDACi Valproate (VPA), sodium butyrate (NaBut) and Trichostatin A (TSA) to modulate induction of the luciferase reporter gene expression under the control of the HPV-16 LCR. Transient transfections were performed in three different immortalized human cell lines: BeWo (trophoblastic cell line), HeLa (cervical cell line) and SiHa (cervical cell line). As shown in Figure 1, all three HDACi increased the luciferase activity, not only in HPV-positive cell lines SiHa and HeLa, but also in the HPV-negative cell line BeWo (Figure 1). We next tested a keratinocyte cell line, HaCaT – a spontaneously-transformed epithelial cell line which does not contain HPV DNA. We chose to use the HDAC inhibitors VPA and TSA, as they had given the strongest induction of the HPV-16 LCR in our initial experiments (Figure 1A, 1B, 1C) and were less toxic to HaCaT cells than NaBut. As seen from the last panel in Figure 1D, VPA and TSA induced several-fold (2-4 fold) the luciferase activity, compared to non-induced control (NI).

### The effect on HPV-16 LCR induction by the HDACi TSA is time-dependent in HaCaT cells

**Figure 2:**
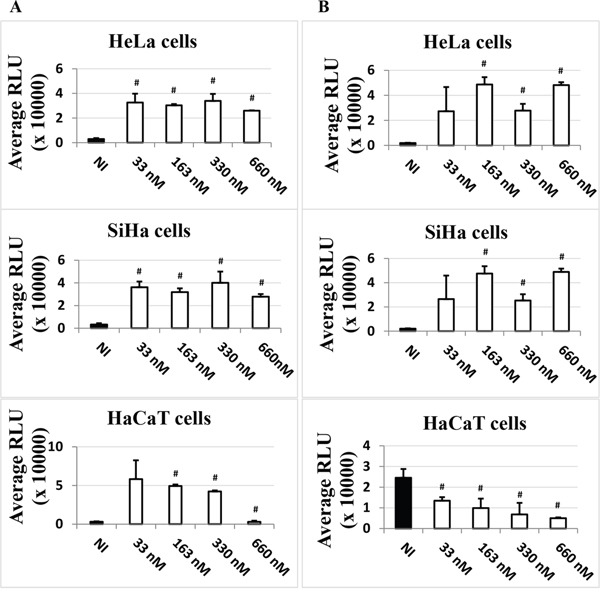
Time-dependent effect of TSA on the LCR transcriptional activity in HeLa, SiHa and HaCaT transformed cell lines Cells were transfected with pLCRluc. TSA (white) was added in the indicated doses either at the time of transfection **A.** or six hours post-transfection **B.** pWtLCRluc-transfected, non TSA-induced (NI, black) cells were used as controls. Twenty-four hours post-transfection, cells were lysed and the luciferase activity was measured in terms of RLU units. These results are the average of three independent experiments done in triplicate. Bars indicate standard deviations. Hash marks above standard deviations indicate statistically significant data, compared to NI.

The data presented so far could indicate an involvement of HDACi in LCR-regulated transcriptional activity. However, these data could also reflect the involvement of HDACi in other host cell modifications, such as differentiation, or additional viral mechanisms but it could also reflect a potential effect on the transfection efficiency. To assess the latter, we compared the impact of adding TSA either at the time of transfection, “T0”, as previously done (Figure 1), with potentially more impact on transfection efficiency, or six hours post-transfection, “T6”, with potentially less impact on transfection efficiency, as the establishment of DNA into cells after transfection generally occurs 4-6 hours post-transfection (ViaFect technical manual). We decided to use TSA as a representative HDACi as it had the strongest inductive effect on the expression of the luciferase gene compared to the other inhibitors, VPA and NaBut (Figure 1). As shown in Figure 2A and 2B, the effect of TSA was comparable at T6 and T0 in HeLa and SiHa cells, increasing strongly the LCR transcriptional activity. This suggested that the activation of the LCR transcriptional response by TSA didn't result from a potential bias of TSA on the transfection efficiency. In HaCaT cells, TSA also strongly increased the LCR transcriptional activity at T0. However, at T6, the drug unexpectedly inhibited in a dose-dependent manner the activity of the HPV-16 LCR (Figure 2B, lower panel). It is worth noting that, in our transfection controls, we could also not detect an effect of the TSA treatment (at T0 or T6) on the transfection efficiency, comparing the pCMV-eGFP transfected HaCaT untreated or TSA treated cells (T0 and T6) by visual inspection under the fluorescence microscope (data not shown).

### Time-dependent effect of TSA on HPV-16 LCR-driven luciferase expression in HaCaT cells is independent of viral early gene expression

**Figure 3:**
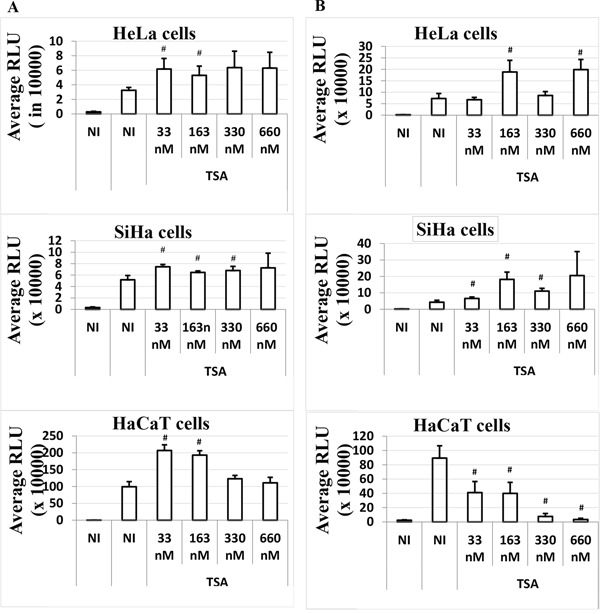
Time-dependent effect of TSA on the LCR transcriptional activity in the presence of HPV-16 early genes in HeLa, SiHa and HaCaT transformed cell lines Cells were transfected as usual with the pWtLCRluc plasmid alone (NI, black), or transfected together with the pLCRearly plasmid (NI, white) and treated with TSA (white). TSA was added either at the time of transfection, T0 **A.** or six hours post-transfection, T6 **B.** Average RLUs were obtained from three independent experiments done in triplicate. Bars represent standard deviations. Hash marks above standard deviation represent statistically significant data, compared to NI.

In order to investigate whether the presence of early proteins in HaCaT cells could suppress the HPV-16 LCR transcriptional inhibition induced by TSA 6 h post-transfection, we expressed all the HPV-16 early proteins under the control of their own promoter, the HPV-16 LCR, in our bioassay system (plasmid pLCRearly). We co-transfected cells with pWtLCRluc (allowing for read-out) and pLCRearly in a 2:1 ratio, and treated the transfected cells with increasing doses of TSA at the time of transfection (T0) or post-transfection (T6). Although HPV-16 early proteins expression strongly induce the LCR transcriptional activity in all the cells (Figure 3), the TSA time-dependent response in HaCaT cells was not influenced by HPV-16 early genes expression.

### TSA treatment of HaCaT cells post-transfection increases integration of the plasmid DNA into the host genome

**Figure 4:**
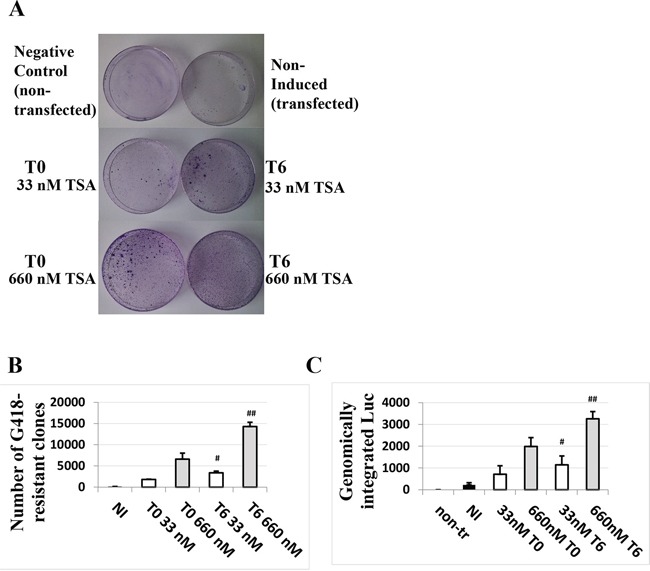
TSA treatment of cells transfected with the HPV-16 LCR causes a dose- and time of induction-dependent integration of the LCR-carrying episomal DNA into the genome of HaCaT cells Cells were transfected with pWtLCRluc and treated with 33 nM or 660 nM TSA at T0 (the time of transfection) or T6 (six hours post-transfection). G 418-resistant clones were stained 10 days post-transfection **A.** and quantified **B.** The average numbers from three independent experiments are presented. Quantitative PCR for the *luc* gene was also performed on the genomic DNA of non-treated cells, transfected but non-induced cells (NI, black) and TSA-treated, transfected cells five days post-transfection in the absence of selection **C.** Presented are the average numbers from three independent experiments done in triplicate. Bars indicate standard deviations. Hash marks represent statistically significant data, comparing the T0 and T6 33 nM-TSA dose (white, single hash marks) and the T0 and T6 660 nM-TSA dose (grey, double hash marks).

The TSA time-dependent response in HaCaT cells suggested a possible effect of TSA on the persistence of the transfected DNA. The impact of TSA treatment on DNA integration in terms of stable transfection efficiency was investigated for the first time. As shown in Figure 4A and 4B, TSA increased the number of G418-resistant clones. The effect was even more pronounced when cells were treated with TSA six hours post-transfection compared to the cells treated with TSA at the time of transfection. We also verified by qPCR the amount of luciferase gene in the HaCaT cells under different conditions and timing of TSA treatment after five days, without selection. The results shown in Figure 4C are in agreement with the results shown in Figure 4A and 4B, suggesting that TSA treatment improves maintenance of transfected DNA, in particular if TSA induction is performed post-transfection.

### TSA induces HaCaT cell differentiation that can be inhibited by the HPV-16 LCR presence at the time of treatment

**Figure 5:**
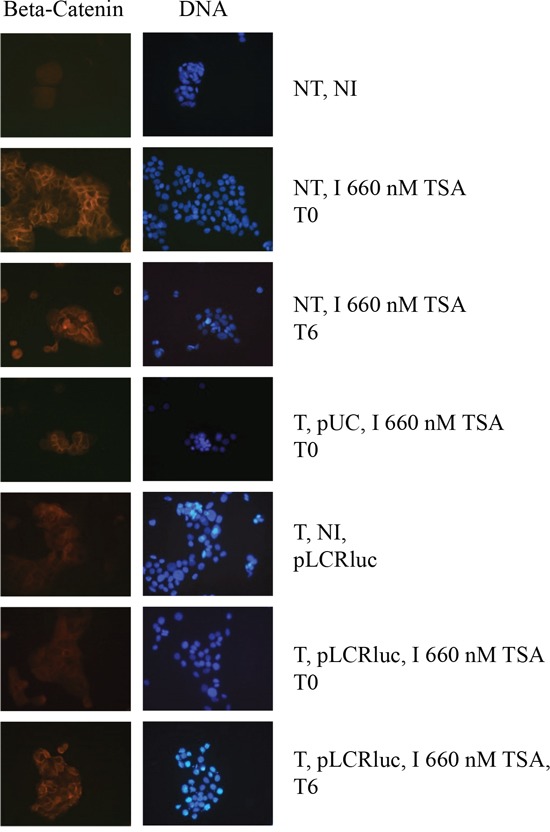
TSA induces the differentiation of keratinocytes, and this differentiation is overcome by the HPV-16 LCR Cells were transfected with pWtLCRluc (T, pLCR, B) and treated with TSA (I) concomitantly with transfection (T0), or six hours later (T6). Non-transfected and non-induced (NT, NI), non-transfected and TSA- induced (NT, I), or cells transfected with pUC-18 (T, pUC, A) were used as controls. Cells were prepared for immuno-fluorescence and photographed.

An important difference between HeLa, SiHa and HaCaT cell lines is that the first two are immortalized, while the latter can differentiate, providing a possible clue to the different HDACi time-dependent responses in these cell lines (Figure 3), as only TSA time-dependent response could be observed in the HaCaT cells. To investigate the potential effect of TSA on HaCaT differentiation, we analyzed the staining of these cells under various conditions using the differentiation marker β-catenin. As shown in Figure 5A, control cells, not transfected and non-TSA treated (NT, NI) or pUC18 transfected and non-TSA treated, are undifferentiated, according to a diffuse cytoplasmic staining of the β-catenin. HaCat transfection had thus no impact on the HaCat cell differentiation. In contrast, cells not transfected but treated with TSA at T0 or T6 or pUC18 transfected and treated with TSA at T0 (Figure 5A) and T6 (data not shown) or pWtLCRLuc transfected and treated with TSA at T6 (Figure 5B) show marks of differentiation, with a defined (“cage-like”) β-catenin staining. This confirmed that TSA promotes the differentiation of keratinocytes, irrespective of the time of treatment or of transfection. However, in pWtLCRLuc transfected HaCaT cells, treated with TSA at T0, surprisingly, we observed no cell differentiation. From these data, we concluded that TSA tends to differentiate keratinocytes, that this effect is specifically reduced by the viral LCR (not observed in pUC18 transfected HaCaT cells) when it is introduced into the cells at the same time as the HDACi treatment and that LCR transcriptional activity is increased by TSA in undifferentiated HaCaT cells (T0) but decreased by TSA in differentiated HaCaT cells (T6) (Figures 3 and 5).

### The HPV-16 LCR transcriptional response to TSA treatment in HaCaT cells is dependent on AP-1 binding sites

**Figure 6:**
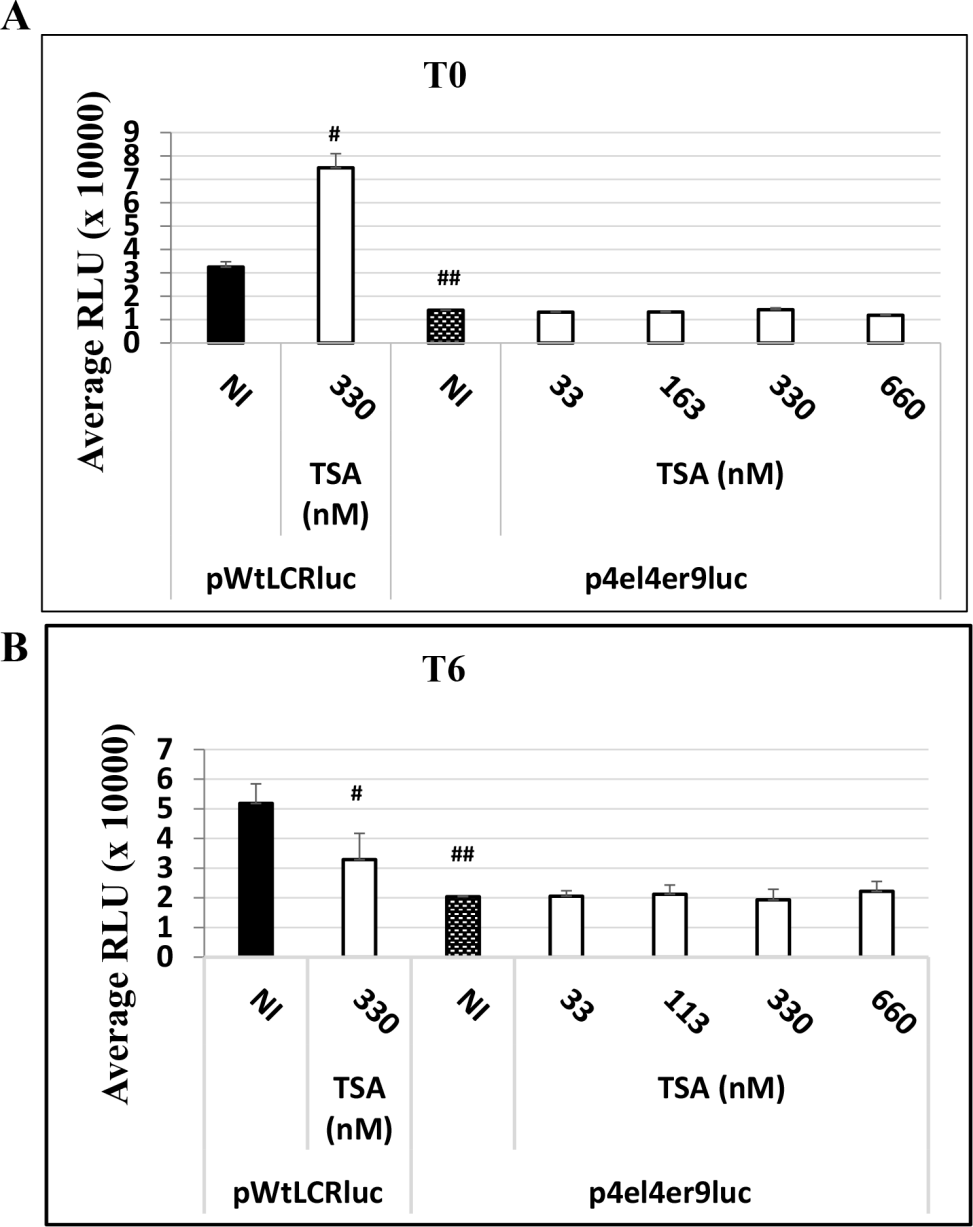
The HPV-16 LCR time-dependent transcriptional response to TSA treatment is dependent on AP-1 binding sites in HaCat cells HaCaT cells were transfected either with the wild-type pWtLCRluc plasmid (NI, black) or with the AP-1 binding sites-mutated plasmid, p4el4er9luc, either untreated (NI, dotted) or treated with TSA at the time of transfection **A.** T0, white for various doses of TSA), or six hours post-transfection **B.** T6, white for various doses of TSA). Luciferase activity was scored in RLU units as usual. The results presented are the averages of three independent experiments done in triplicate. Bars indicate standard deviations. Hash marks above standard deviations represent statistically-significant data, compared to NI pWtLCRluc (black).

As the HPV-16 LCR contain many different binding sites for homo- or hetero-dimers transcription factors (AP-1 and C/EBPβ transcription factors), we studied the involvement of the 3 major AP-1 binding sites of the HPV-16 LCR on the observed TSA effect in transfected HaCat cells. For this purpose, we compared the luciferase activity in HaCaT cells transfected either with the wild type pWtLCRluc plasmid or with the mutated AP-1 derived pWtLCRluc plasmid, p4el4er9luc, in which all three major AP-1 binding sites in the LCR region of HPV-16 have been mutated [32]. In the absence of TSA treatment, we observed a marked luciferase activity decrease in cells transfected with the AP-1 mutant construct, consistent with the reported role of AP-1 in HPV LCR-driven transcription. Interestingly, addition of increasing doses of TSA (at T0 or T6) to the p4el4er9luc – transfected cells, failed to reproduce the effects observed on HaCaT cells transfected with its wild-type counterpart and treated with the same TSA doses (Figure 6). The three major AP-1 binding sites are thus required for the effects of TSA on the transcriptional activity of the HPV-16 LCR.

## DISCUSSION

It is a general opinion in the scientific literature that HDACi could be used as potential drugs to cure cervical cancer. By virtue of their ability to induce differentiation, growth arrest and apoptosis in several cancer cell line models, these agents were introduced into clinical trials [29]. Prominent studies by Finzer *et al.* have shown that HDACi (e.g. NaBut, phenylbutyrate and TSA) can arrest cells at the G1 to S transition of the cell cycle in the HPV-positive HeLa cell line [19, 20]. Finzer *et al.* have also demonstrated that HDACi induce an intrinsic type of apoptosis in HPV-positive cells [18]. In a subsequent study, Darvas *et al.* additionally showed that HDACi sensitize resistant HeLa cells to receptor-induced cell death [17]. Despite these insights, little is known about the impact of these agents in the context of the HPV life cycle and its outcome on HPV infection. This is particularly important, as it is known that most cervical lesions, although caused by infection with only one type of HR HPV, are typically surrounded by co-infections with multiple viruses [33, 34]. In an effort to study these issues in more detail, we first evaluated the impact of several HDACi on the HPV-16 LCR-driven transcription in various cell lines. We demonstrated that TSA, VPA and NaBut induce several-fold the transcription of the *firefly* luciferase reporter gene under the control of the HPV-16 LCR in the cervical cell lines HeLa and SiHa, but also in the HPV-negative trophoblastic cell line BeWo. Furthermore, TSA and VPA induced a 2-4-fold increase in the LCR-driven transcription of the luciferase reporter in HaCaT cells, an HPV-negative keratinocyte cell line able to differentiate (unlike HeLa, SiHa and BeWo cells). This is in agreement with Bechtold et al. reporting that E6/E7 mRNA production was upregulated by TSA from an episomal viral DNA in the W12 keratinocyte cell line [31]. The accessibility of the HPV-16 LCR to the numerous transcriptional HPV-16 LCR activators, able of cross-coupling, such as AP-1 and NF-IL6/C/EBPβ, could be, as expected, improved [35, 36]. Some of these transcriptional activators, for instance AP-1, has been reported to interact with HAT or HDAC and to regulate positively or negatively promoters [37, 38]. We previously observed that c-jun protein can cooperate with CBP/p300 general coactivators to activate the HPV-16 LCR [39] and Stiehl *et al*. reported that CBP/p300, interacting with various transcription factors but also with regulatory proteins such as HAT, can be autoacetylated [40]. This autoacetylation can in turn regulate its own HAT activity and can be affected by TSA, increasing its dynamic transcriptional activity [40]. The HPV-16 LCR can also be activated by C-EBPβ through a multiple c/EBPβ binding sites present in the LCR [39] and interestingly the transcriptional activity of this factor has also been reported to be activated by p300 acetylation [41]. So, it is tempting to think that the TSA treatment could have positively regulated the HPV-16 LCR activity through the activation of the p300/CBP which in turn activated various transcription factors binding multiple binding sites.

To assess the impact of HDACi on the transcriptional activity of LCR-controlled genes directly, excluding possible effects of these agents on other host cell and viral modifications – e.g. cell differentiation or viral DNA maintenance – or on the transfection efficiency, we evaluated the effect of adding TSA concurrently with transfection, or conversely, six hours post-transfection. According to our results, TSA had a clear “boosting” effect on the HPV-16 LCR-driven transcriptional activity in HeLa and SiHa cells, both when TSA treatment was performed at the time of transfection or post-transfection. The fact that TSA could still strongly activate the LCR transcriptional activity even added 6 hours post-transfection alleviated the doubt that this activation could have been linked to transfection efficiency. However, surprisingly, although TSA increased the LCR transcriptional activity in HaCaT cells when added at T0, added six hours post-transfection it clearly reduced the LCR transcriptional activity, independently of HPV early gene expression. This suggested an additional effect of TSA in HaCaT cells on either the host cell or the transfected DNA – e.g. cell differentiation or DNA integration, as confirmed later. Indeed, HeLa cells and SiHa cells are immortalized, unable to differentiate but HaCaT cells treated with TSA showed marked signs of differentiation, notably when TSA was added 6 hours post-transfection (T6) and that it reduced the LCR transcriptional activity. This effect of TSA on the keratinocyte differentiation is in agreement with previous results from Markova *et al.* on cultured epidermal explants treated with TSA [42] and the HDACi effects on differentiation was already documented on various cells [43, 44]. It is worth noting that TSA-induced differentiation seemed specifically inhibited when pWtLCRLuc plasmid was transfected at the time of the TSA treatment (T0), when TSA is able to activate the LCR promoter, suggesting a signaling competition between the differentiation program and the LCR transcriptional activity. Indeed, the artificially high number of pWtLCRluc copies introduced into the HaCaT cells at T0 during transient transfection could “titrate away” some transcription factors required for HaCaT cell differentiation, while at T6 the amount of available plasmids could have already dropped, as expected from a transient transfection and as observed in our integration analysis by qPCR. Durst *et al.*, have shown that HPV early gene expression is inversely related to the differentiation of the host keratinocyte [45]. Various differentiation-regulated transcriptional factors could be involved, as many of those (e.g. AP-1, YY-1, CDP/Cut, NF-IL-6/C-EBPβ) can bind and regulate the HPV-16 LCR [35, 36, 45–47]. AP-1 and YY1 transcription factors have been shown to bind and regulate, positively or negatively, respectively, both the HPV-16 LCR but also genes promoters involved in differentiation [37, 47–50]. As Wang *et al.* showed that acetylation of dimeric AP-1 complexes enhanced their binding to HPV-16 DNA [35], hyper-acetylated conditions in HaCaT cells during treatment of transfected cells with TSA at time zero, could for example preferentially promote the binding of transcription-activating AP-1 complexes such as Fra-2/AP-1 to the HPV-16 LCR, which in turn would not be anymore “available” to bind and regulate promoters involved in the differentiation genes expression. This hypothesis was partly investigated comparing the effect of TSA on the luciferase activity of HaCat cells transfected either with the pWtLCRLuc plasmid or with a mutated plasmid lacking the 3 major AP-1 binding sites. The lack of LCR upregulation by TSA at T0 and LCR downregulation by TSA at T6 when using the AP-1 binding sites mutated construct confirmed that transcriptional factors able to interact with at least one of the three major AP-1 binding sites of the HPV-16 LCR are indeed involved in those regulations. It is worth noting that we could not detect any effect of TSA on the luciferase activity when HaCaT cells were transfected with a classic artificial pAP-1-Luc reporter plasmid (luciferase gene expression under the control of tandem AP-1 binding sites in front of a minimal promoter), suggesting that other element(s) in the HPV-16 LCR (1000 pb between nt 7007 and nt 117 of the viral genome) are also necessary (data not shown).

Another factor responsible for the temporal transcriptional response to TSA treatment in HaCaT cells could be linked to the physical state (extra-chromosomal/integrated) of the pWtLCRluc transfected DNA. To our knowledge, this is the first study on the effect of TSA treatment on the integration efficiency of extra-chromosomal DNA. We show that, both in experiments studying stable transfection efficiency, as well as, in qPCR experiments, TSA treatment of HaCaT cells promotes an improved maintenance of transfected DNA in the cell. As we used a plasmid unable to replicate without viral early protein expression and as 5-10 days post-transfection, only integrated DNA can be maintained in the cells, our results clearly suggest that HDACi promoted extra-chromosomal DNA integration. HDACi are known to induce chromatin decondensation, increasing DNA exposure, but are also known to downregulate the activity of the DNA repair machinery [51–53]. It is therefore tempting to think that this might have also promoted the integration of extra-chromosomal DNA. Considering that Bechtold *et al.*, have shown that TSA boosts transcription of the E6 and E7 early genes from episomal templates (in agreement with our data), while suppressing it from intra-chromosomal viral templates [31], it is possible that the decrease in transcription efficiency of the HPV-16 LCR, promoted by TSA treatment post-transfection, could be partially due to the TSA-promoted increase of integrated copies of the pWtLCRluc in HaCaT cells. However, we think that the impact of TSA on HaCaT differentiation, is more important than the role of TSA on the integration DNA status in reducing LCR-driven transcription. In the HIV field, HDACi have also entered clinical trials [22]. While combinatory antiretroviral therapy (CART) is capable of durably suppressing viremia in HIV-infected individuals, it is unable to cure infection. The best understood barrier to eradicating HIV infection is the persistence of a latent reservoir of infected resting memory CD4+ T cells. By virtue of their quiescent state, these cells are not thought to express HIV-1 antigens, rendering them insensitive to CART and able to escape the host immune system. HDACi are well-known inducers of transcription from the HIV-1 promoter [22, 26–28]. As such, these drugs are currently tested in combination with CART for their abilities to induce viral expression in the resting CD4+ T-cell reservoir in order to trigger immune-mediated clearance of infected cells. Given that both the HIV-1 and HPV promoters are regulated by similar transcription factors (e.g., C/EBPβ, AP-1, NF-κB, YY-1) [35, 36, 49, 50, 54–58], it is not surprising that transcription from both viral promoters can be induced by HDAC inhibition, unless, as shown in our study, HDACi promote differentiation and/or viral DNA integration. In conclusion, our results suggest that HDACi could affect the integration of viral extra-chromosomal DNA into the genome of the host cells, but also engender a dangerous episomal burst of transcription from the HPV viral promoter in undifferentiated cells. The molecular mechanisms elucidating these events will be the subject of further studies. Our results therefore underscore the need to carry out further experiments, especially *in vivo* in mouse models or with clinical samples, to better evaluate the impact of the use of HDACi in the treatment of HPV infection.

## MATERIALS AND METHODS

### Plasmids

The construction of the pLCR early (or pHPV16 early) plasmid containing the HPV16 early genes under the control of the HPV16 LCR cloned in the pCR-XL-TOPO vector, has been previously described [32]. The pWtLCRluc plasmid containing the HPV16 LCR in front of the firefly luciferase gene in pUHC 13.3 was also previously described [54]. The AP-1 binding sites mutant plasmid, p4el4el9luc, was obtained by site-directed mutagenesis of pWtLCRluc, [32]. The peGFP-NLS was a kind gift of X. Saelens (Molecular Virology, VIB Department for Molecular Biomedical Research, Ghent University, Belgium) and the pAP-1-Luc was a kind gift of Y. Jacob (Virus interactomics, Institut Pasteur de Paris, France).

### Cell culture, transfections and drug treatment

The trophoblastic BeWo cell line, keratinocyte HaCaT cell line, and HeLa (HPV-18-harbouring) and SiHa (HPV-16-harbouring) immortalized cervical epithelium cell lines were maintained in DMEM (Gibco) supplemented with 10% fetal calf serum (Gibco), 1% penicillin/streptomycin and 1% Non-Essential Amino Acids (Lonza). Transfections of HeLa and SiHa cells were carried out by the calcium phosphate precipitation method, as described previously [54, 59]. Transfections of BeWo cells were done using the jetPEI transfection reagent (Polyplus-transfection Inc), according to the manufacturer's specifications. HaCaT cells were transfected using the ViaFect transfection reagent (Promega), again according to the manufacturer's specifications. The HDACi Valproate (VPA), sodium butyrate (NaBut) and trichostatin A (TSA) were a kind gift from Dr. Carine van Lint (ULB). Serial dilutions in serum-free DMEM were used to prepare fresh aliquots of each inhibitor at the desired range of concentrations and cells were treated at the time of transfection (T0) or six hours post-transfection (T6), as required. Parallel transfection using the peGFP-NLS reporter plasmid allowed us to assess the transfection efficiency in our experiments by direct fluorescent cell visualization under the fluorescence microscope. A transfection efficiency of 40-60% was routinely achieved.

### Measurement of luciferase activity

Cells were transfected and HDACi-treated as described above. To assess for transfection efficiency, cells were transfected in parallel with peGFP-NLS. A transfection efficiency of 40% to 60% (assessed by visual inspection of the cells under the fluorescent microscope) was routinely achieved. Luciferase activity was measured 24 hours post-transfection using the Luciferase Assay System (Promega). Briefly, 100 μL Luciferase Assay Reagent was injected into 20 μL cell lysate per transfected sample. Relative Luminescence Units (RLU) were measured for 60s using a luminometer (Berthold Technologies, Germany). Luciferase activity was normalized to the total protein content of the cell lysates, as determined by the Bradford reagent (Sigma-Aldrich). The results shown are representative of at least three different experiments using different DNA preparations.

### Assessment of genomic integration of pWtLCRluc: G418 selection and qPCR

HaCaT cells were transfected with pWtLCRluc together with pCMVeGFP (carrying the *Neomycin-*resistance gene; ratio 10:1) and HDACi-treated in six-well plates. Twenty-four hours post-transfection, 50,000 cells per each condition were transferred into 10 cm-plates and further incubated with G-418 to a final concentration of 500 μg/mL. Selection continued for 10 days, with addition of fresh G-418-containing medium every 2-3 days. At the end of the selection period, cells were washed with PBS, fixed with 100% ice-cold methanol, and stained with Crystal violet. G-418-resistant clones were photographed and counted on a bacterial cell counter. For the qPCR, five days post-transfection with pWtLCRluc, cells were washed with the CellScrub washing buffer (Genlantis) in order to remove cationic lipid/DNA complexes associated with the cell surface after transfection. Total DNA was extracted using the DNA Realeasy reagent (Nippon Genetics), according to the manufacturer's specifications. To discriminate between integrated and input episomal pWtLCRluc copies, and to normalize for transfection efficiency, isolated DNA was digested with *DpnI* (digests dam methylated bacterial DNA). Samples were purified with phenol-chloroform-isoamyl alcohol (25:24:1), ethanol-precipitated, washed with 70% ethanol, dried and resuspended in TE. The primers used to amplify the *firefly* luciferase reporter gene have already been described [32]. qPCR was performed using the IQ SYBR Green Supermix (BioRad), on the BioRad Thermal Cycler, under the following conditions: a DNA polymerase activating step at 95°C for 3 minutes, followed by 40 cycles of denaturation at 95°C for 1 minute and annealing/elongation at 60°C for 30 seconds. As reference genes, βActin and GAPDH were also amplified simultaneously with the *firefly* luciferase gene, and the relative copy number of genomically integrated pWtLCRluc was calculated by the ΔΔCt method. The primers for amplifying the two reference genes have already been described [32].

### Immunolabelling for fluorescence microscopy

HaCaT cells were plated on 8-well slides and transfected and HDACi-treated as described already. At 24 hours post-transfection, cell preparations were rinsed with PBS and fixed with 4% paraformaldehyde (PAF) for 20 min. After several more washes with PBS and in PBS/0.01% triton X-100, non-specific sites on cells were blocked with a 20-times dilution of normal donkey antibody and the slides were left at room temperature for 1 hour. After this, a 100-fold dilution of mouse anti-β-catenin antibody (Santa Cruz Biotechnologies) was applied to the cells and slides were left to incubate in a humid chamber for 24 hours at 4°C. All further steps were carried out in the dark. Namely, after two washes in PBS, cell preparations were incubated in a 100-fold dilution of donkey-anti-mouse Cy3 (Vector laboratories) secondary antibody, for 30 minutes at room temperature. After that, washed labelled cells were mounted with Prolong Gold antifade reagent with DAPI (Invitrogen). A week later, dried slides were visualized by fluorescence microscopy.

### Statistics

The data presented are representative of at least three independent experiments done in triplicate using different DNA preparations. Statistical analyses were performed using MS Office software. Statistical differences were determined by using a two-tailed, unpaired Students’ t-test. A *p* value of less than 0.05 was considered as statistically significant.
